# Mediating effect of working conditions on the association between education and early labour market exit: a cohort study of Swedish men

**DOI:** 10.1136/oemed-2024-109594

**Published:** 2024-11-25

**Authors:** Emma Carlsson, Tomas Hemmingsson, Melody Almroth, Daniel Falkstedt, Katarina Kjellberg, Emelie Thern

**Affiliations:** 1 Global Public Health, Karolinska Institutet, Stockholm, Sweden; 2 Public Health Sciences, Stockholm University, Stockholm, Sweden; 3 Institute of Environmental Medicine, Karolinska Institutet, Stockholm, Sweden; 4 Centre for Occupational and Environmental Medicine, Stockholm County Council, Stockholm, Sweden

**Keywords:** Public health, Longitudinal studies, Men, Aging

## Abstract

**Objectives:**

It is not fully known what explains educational inequalities in early labour market exits. This study aims to examine the mediating effect of exposure to unfavourable working conditions, measured by low job control and high physical workload, on the association between education and early labour market exit among men.

**Methods:**

This register-based study included all men born 1951–1953, who underwent Swedish military conscription in late adolescence and had a registered educational level in 2005 (n=115 998). These men were followed from ages 53–55 to 64 regarding early labour market exit (disability pension, long-term sickness absence, long-term unemployment, early old-age retirement with and without income). Mediation analysis was used to examine the role of job control and physical workload in explaining the educational differences in early exit. Factors measured in childhood and late adolescence were included as confounders.

**Results:**

The proportion mediated by job control was around 17% and for physical workload around 22% for the least educated men for exit through disability pension, long-term sickness absence and long-term unemployment. For early old-age retirement with and without income, working conditions were not mediating factors, except for job control mediating up to 18% for exit through early old-age retirement with income.

**Conclusions:**

Job control and physical workload seem to be important factors explaining the educational differences in most early exit routes, also after accounting for early life factors. These results indicate the importance of improving working conditions to decrease inequalities in early labour market exit and prolong working life.

**WHAT IS ALREADY KNOWN ON THIS TOPIC**

- Lower educational attainment is a risk factor for leaving the labour market earlier than normative retirement age. However, the mechanisms behind the educational inequalities seen in early labour market exits are not fully known.

**WHAT THIS STUDY ADDS**

- This study found job control and physical workload to be important mediating factors for educational differences in early labour market exit through five different exit routes.

**HOW THIS STUDY MIGHT AFFECT RESEARCH, PRACTICE OR POLICY**

- The results indicate the importance of improving working conditions to decrease inequalities in early labour market exit and increase later-life labour force participation.

## Introduction

 Labour force participation in older working ages has gradually increased in Europe during the past decade.^1^ This is in part explained by the reformed pension systems implemented with the goal to raise the retirement age, due to population ageing.^2^ However, even in Sweden, with a comparatively high labour force participation rate among older working ages, almost 20% of the population aged 55–64 did not participate in the labour force in 2022.^3^ In the past, disability pension was the most common way to exit the labour market before the age 65 in Sweden. However, social insurance reforms have increased the use of alternative early exit routes, such as taking old-age pension.^4^

Lower socioeconomic position (SEP) is a risk factor for early labour market exit.^5^,^9^ Lower educated individuals are more often forced out involuntarily due to, for example, poor health or unemployment, compared with higher educated individuals.^9^ The mechanisms behind the SEP differences in early exit are not fully discovered and it is important to examine potential explanatory factors to prevent early labour market exits. This is especially important for the less educated who have a considerably higher risk of early labour market exit.^5^,^9^

Previous research has found that factors from early life, such as childhood SEP, cognitive ability and health seem to be important when explaining educational differences in early labour market exit.^10^,^12^ This selection effect partly determines both educational attainment, occupational success and later health.^13^ However, these early factors do not fully explain the association between level of education and early labour market exit,^10^,^12^ especially not for exit through early old-age retirement,^10 12^ and mediating factors, present after selection into education and occupation, may also be of importance.

Occupational factors may play an important role in the educational differences in early labour market exit as they are risk factors for leaving the labour market early.^14^,^16^ Unfavourable working conditions are not randomly distributed in the population, but rather, these are more often found among those with lower SEP^16 17^ and lower levels of education.^718^,^21^ Unfavourable working conditions could include, for example, low job control (where individuals lack influence over their work) or high physical workload (involving heavy lifting, repetitive work or awkward body postures).^14 15^ Low job control is associated with an increased risk of disability pension,^7 14 22^ sickness absence,^23 24^ unemployment^25^ and early old-age retirement.^22 25^ On the other hand, experiencing high job control is associated with later retirement and prolonged working life.^26^ Experiencing a high physical workload has previously been linked to a higher risk of receiving disability pension,^7 15 16 22 27^ sickness absence^15 23 28^ and unemployment.^16 18^ However, the association between high physical workload and early old-age retirement remains unclear with research yielding mixed results. While some studies show no or weak associations,^7 22^ others have demonstrated a positive association.^15^ Hence, both job control and physical workload are working conditions associated with early labour market exit. Furthermore, job control seems to better predict early exit compared with other psychosocial factors.^14 29^

So far, only a limited number of previous studies have examined the role of working conditions on the association between education and early labour market exit.^718^,^21^ A study found that low job control, low rewards and high physical demands at work mediate the association between level of education and disability pension.^7^ However, these work factors only played a minor role in exit through unemployment and had no impact on economic inactivity.^7^ Another study found that physical demands, quantitative demands and influence at work did not have an important mediating role in explaining educational differences in disability pension.^18^ However, they found that physical demands and influence at work, but not quantitative demands, were important in explaining educational differences in unemployment.^18^ Nevertheless, the results from previous research are inconclusive regarding the importance of the role of working conditions in explaining the educational differences in early labour market exit. Furthermore, these previous studies have some important methodological issues. Only two studies included more than one exit route,^7 18^ some studies were not able to follow the participants up until 65 years of age^18^,^20^ and two of these studies were published more than a decade ago and may, therefore, not represent current conditions.^20 21^

Therefore, the present study aims to examine the mediating effect of exposure to unfavourable working conditions, measured by low job control and high physical workload, on the association between education and early labour market exit among men.

## Methods

This register-based cohort study included all men born in 1951–1953 who went through the conscription examination for military service in Sweden at the age of 18–20 years (n=167 499). At the time, the conscription examination was obligatory by law for the whole Swedish male population aged 18–20 years and included at least 90% of the population.^30^ This study includes all men alive and registered in Sweden in the year 2006. Men who died before 2006, who received disability pension before 2006, who did not have information on education or who did not have any registered job title in 2005, were excluded from the analysis. Excluded men had lower educational levels (for those who did not have missing information on education), lower childhood SEP and worse health in late adolescence (online supplemental table 1). The final analytical sample consisted of 115 998 men (see online supplemental figure 1).

### Level of education (exposure)

Educational attainment was obtained the year 2005 from the Longitudinal Integrated Database for Health Insurance and Labour Market Studies (LISA).^31^ The highest level of attained education was used and categorised into five separate groups: ≤9 years (primary school), 10–11 years (1–2 years of upper secondary), 12 years (3 years of upper secondary), 13–14 years (1–2 years of university) and ≥15 years (at least 3 years of university). The last group served as the reference group. This categorisation was used to capture the graded relationship between education and early labour market exit seen in previous research.^10^,^12^

### Early labour market exit (outcome)

Early labour market exit was measured from 1 January 2006 (when the men were 53–55 years old) up until 31 December the year the subject turned 64 years old (the year 2017 at the longest). Information on early exit was collected from the LISA-register. Five different early exit routes were defined: disability pension, long-term sickness absence, long-term unemployment, early old-age retirement without income and early old-age retirement with income. Disability pension can, in Sweden, be granted by the Swedish Social Insurance Agency to all individuals aged 30–65 years with a medically verified disease or injury and a permanently reduced work capacity by at least 25%.^31^ In this study, disability pension was defined as all men receiving full-time or part-time disability pension benefits. Long-term sickness absence was defined as receiving sickness benefits for at least 90 annual days during two consecutive years from the Swedish Social Insurance Agency. Disability pension in Sweden is granted for those with a permanently reduced work capacity, whereas sickness benefits are given as a temporary benefit. Long-term unemployment was defined as being registered as full-time unemployed at the Swedish Public Employment Service for at least 180 annual days during two consecutive years. Both long-term sickness absence and long-term unemployment were defined to capture men further away from being active in the labour market, as in our previous research,^10^ compared with shorter periods of sickness absence or unemployment. Early old-age retirement in Sweden can be granted from 61 years of age. There are no restrictions on how much income from work one can have while still withdrawing a pension. In this study, early old-age retirement without income was defined as receiving any old-age pension during 1 year and having an income below one Price Base Amount (PBA)^32^ (approximately €4500 per year) the following year, while early old-age retirement with income was defined as receiving any old-age pension during 1 year and having an income above one PBA the following year. In the analyses of early old-age retirement without and with income, men who died or received disability pension between the year 2006 (at ages 53–55) and age 61 were excluded (n=5406).

### Working conditions (mediators)

Working conditions were included by estimating job control and physical workload during 2005 (when the men were 52–54 years), using job exposure matrices (JEMs). The JEMs are based on the Swedish Work Environment Surveys, based on 90 000 responses, which were conducted between 1997 and 2013 and include 355 occupations.^29 33^ Through the JEMs, occupational codes are classified into different levels of exposure to job control and physical workload, as described previously.^33 34^ Job control at work was measured by seven survey questions on a 5-point scale, including questions about the individual’s ability to decide when to take breaks, how to structure their work and opportunities for learning and development of skills. Physical workload at work was measured by eight survey questions on a 5-point or 6-point scale, including questions about heavy lifting, repetitive work, and physically demanding work. In the JEMs, a gender-specific mean value index was created for each of the 355 occupations, for job control and physical workload separately. These mean index values of the JEMs were linked to the men in this study by their registered occupation in 2005. For this study, after linking the index values to the men’s occupation, both job control and physical workload were dichotomised, comparing the 25% with the lowest job control and highest physical workload, respectively, to the rest of the study population.

### Childhood and late adolescence (confounders)

Factors measured in childhood and late adolescence were included as these seem to be important contributors to educational differences in early labour market exit, according to previous research.^10^,^12^ These factors may act as confounders in the associations between education and early labour market exit and between the working conditions and early exit (see online supplemental figure 2).

Childhood SEP was measured by parental educational attainment. This information was obtained from the National Population and Housing Census from the year 1970 (when the men were 17–19 years old). The value from the parent with the highest level of education was used and education was classified into the same five categories as the exposure.

Factors measured at the conscription examination include; cognitive ability, stress resilience, body mass index and psychiatric and musculoskeletal diagnoses (described in online supplemental file). The conscription examination consisted of extensive testing, both physical and psychological, described in detail elsewhere.^30^

Missing values on the confounders were coded as separate categories since analysing complete cases yielded similar results (online supplemental tables 2 and 3). Missing data were due to a lack of data in the registers and internal missing data.

### Statistical analysis

Differences in baseline characteristics by educational level were tested using Pearson’s χ^2^ tests. A generalised linear model with a log-binomial regression was used to test the association between the two mediation variables, job control and physical workload, and each outcome, respectively. Since each model was run separately for each outcome, an individual could be observed in several different outcomes.

The mediating effect of the working conditions was analysed using the potential-outcomes framework to decompose the total effect of education on the outcome into direct and indirect effects.^35^ The direct effect captures the effect of education on the outcome without passing through the mediator, while the indirect effect captures the effect of education on the outcome through the mediator^36^ (see online supplemental figure 2). The mediation analysis was performed using the mediate command in Stata V.18. The mediate command fits a model for each exit route and both working conditions separately. These models were specified as logistic models and risk ratios (RRs) are presented with 95% CIs, by educational level. For each outcome and mediator, a crude model and a model adjusted for all factors measured in childhood and late adolescence are presented. The proportion of the total effect that is due to the indirect effect is presented as proportion-mediated (the indirect effect multiplied by the direct effect gives the total effect). All men who died or emigrated during the follow-up were included in the main analysis, but a sensitivity analysis was done after excluding these men.

Since previous research is unclear on which method is more suitable for mediation analysis,^37^ we included a robustness test for the mediation analysis. For that purpose, a model using Cox proportional-hazards regressions when adjusting for the confounders and the mediators on the association between education and early labour market exit was used (see online supplemental table 4 for results and a full description of this analysis).

All analyses were performed by using Stata Statistical Software: Release V.18 (StataCorp).

## Results

During the follow-up period, 5772 (5%) men exited the labour market through disability pension, 9442 (8%) men exited through long-term sickness absence, 4764 (4%) men exited through long-term unemployment, 31 089 (28%) men exited through early old-age retirement without income and 19 724 (18%) men exited through early old-age retirement with income. The average age at exit through disability pension, long-term sickness absence and long-term unemployment was 58 years, and for early old-age retirement with and without income, the average age at exit was 62 and 63 years, respectively.

Men with lower levels of education also had lower childhood SEP and worse health in late adolescence (see table 1). Furthermore, men with lower levels of education more often experienced low job control and high physical workload compared with the higher educated.

**Table 1:** Baseline characteristics of study population, stratified by years of education

Years of education	≥15 n (%)	13–14 n (%)	12 n (%)	10–11 n (%)	≤9 n (%)	P value
Total	21 310 (18.4)	17 399 (15.0)	16 898 (14.6)	35 187 (30.3)	25 194 (21.7)	
Childhood variable						
Parental education						<0.001
≤9	7731 (36.3)	8955 (51.5)	9675 (57.3)	23 379 (66.4)	19 160 (76.0)	
10–11	4150 (19.5)	3588 (20.6)	3310 (19.6)	6267 (17.8)	2868 (11.4)	
12	2653 (12.4)	1935 (11.1)	1474 (8.7)	1985 (5.6)	795 (3.2)	
13–14	1492 (7.0)	807 (4.6)	508 (3.0)	605 (1.7)	238 (0.9)	
≥15	3788 (17.8)	1036 (6.0)	772 (4.6)	445 (1.3)	165 (0.7)	
Missing	1496 (7.0)	1078 (6.2)	1159 (6.9)	2506 (7.1)	1968 (7.8)	
Late adolescence variables						
Cognitive ability						
High (7–9)	12 577 (59.0)	7714 (44.3)	5792 (34.3)	5262 (14.9)	2115 (8.4)	<0.001
Medium (4–6)	7052 (33.1)	8081 (46.5)	8758 (51.8)	20 500 (58.3)	12 918 (51.3)	
Low (1–3)	460 (2.2)	820 (4.7)	1527 (9.0)	7562 (21.5)	8663 (34.4)	
Missing	1221 (5.7)	784 (4.5)	821 (4.9)	1863 (5.3)	1498 (5.9)	
Stress resilience						
High (7–9)	7334 (34.4)	5441 (31.3)	4441 (26.3)	6268 (17.8)	3642 (14.5)	<0.001
Medium (4–6)	9910 (46.5)	8942 (51.4)	9178 (54.3)	19 761 (56.2)	13 423 (53.3)	
Low (1–3)	2770 (13.0)	2176 (12.5)	2408 (14.3)	7173 (20.4)	6539 (25.9)	
Missing	1296 (6.1)	849 (4.8)	871 (5.1)	1985 (5.6)	1590 (6.3)	
BMI≥25	701 (3.3)	835 (4.8)	868 (5.1)	2281 (6.5)	2033 (8.1)	<0.001
Missing	1870 (8.8)	1325 (7.6)	1279 (7.6)	3109 (8.8)	2225 (8.8)	
Psychiatric diagnoses	1945 (9.1)	1490 (8.6)	1591 (9.4)	4645 (13.2)	4126 (16.4)	<0.001
Musculoskeletal diagnoses	3208 (15.1)	2718 (15.6)	2628 (15.6)	5783 (16.4)	4252 (16.9)	<0.001
Working conditions						
Job control—low	986 (4.6)	1789 (10.3)	3426 (20.3)	11 486 (32.6)	11 188 (44.4)	<0.001
Physical workload—high	602 (2.8)	1176 (6.8)	3470 (20.5)	12 323 (35.0)	10 898 (43.3)	<0.001
Outcome						
Disability pension	610 (2.9)	603 (3.5)	739 (4.4)	2127 (6.0)	1693 (6.7)	<0.001
Long-term sickness absence	1028 (4.8)	1061 (6.1)	1241 (7.3)	3530 (10.0)	2582 (10.3)	<0.001
Long-term unemployment	595 (2.8)	646 (3.7)	696 (4.1)	1726 (4.9)	1101 (4.4)	<0.001
Early old-age retirement without income	4277 (20.7)	4664 (27.7)	4 827 (29.8)	9939 (29.9)	7382 (31.3)	<0.001
Early old-age retirement with income	3077 (14.9)	2983 (17.7)	2755 (17.0)	6300 (18.9)	4609 (19.5)	<0.001

BMIbody mass index

Low job control was associated with a higher risk of leaving the labour market early through all five exit routes (table 2). High physical workload was associated with a higher risk of leaving the labour market through all early exit routes except early old-age retirement with income (and, therefore, mediation analysis using physical workload as mediator was not conducted for this outcome). Instead, high physical workload had a small protective effect against exit through early old-age retirement with income.

**Table 2:** Unadjusted risk ratios (RR) and 95% CI for the association between low job control and high physical workload and each outcome separately, among the total study population

	Disability pensionRR (95% CI)	Long-term sickness absenceRR (95% CI)	Long-term unemploymentRR (95% CI)	Old-age retirement without incomeRR (95% CI)	Old-age retirement with incomeRR (95% CI)
Job control—low	1.67 (1.59 to 1.76)	1.55 (1.49 to 1.61)	1.33 (1.26 to 1.42)	1.04 (1.02 to 1.06)	1.14 (1.11 to 1.17)
Physical workload—high	1.66 (1.58 to 1.75)	1.60 (1.53 to 1.66)	1.40 (1.32 to 1.48)	1.11 (1.08 to 1.13)	0.93 (0.90 to 0.96)

Unadjusted RRs using low job control and high physical workload, where the 25% with the lowest job control and highest physical workload, respectively, are compared tocompared with the rest of the study population.

Men with lower levels of education had a higher risk of leaving the labour market early through all exit routes compared with the higher educated (see total effects in tables3^5^). RRs for the least educated compared with the highest educated men were 2.35 (95% CI 2.14 to 2.57) for disability pension, 2.12 (95% CI 1.98 to 2.28) for long-term sickness absence, 1.57 (95% CI 1.42 to 1.73) for long-term unemployment, 1.51 (95% CI 1.46 to 1.56) for old-age retirement without income and 1.31 (95% CI 1.26 to 1.37) for old-age retirement with income, in the crude models.

**Table 3:** Mediation analysis with decomposition of the effect of level of education (in years) and early exit through disability pension, long-term sickness absence and long-term unemployment, into total effect, direct effect and indirect effect using job control as mediator

Years of education	13–14RR (95% CI)	%∆	12RR (95% CI)	%∆	10–11RR (95% CI)	%∆	≤9RR (95% CI)	%∆
Disability pension (5772 events, 4.98%)								
Model 1—crude								
Total effect	1.21 (1.08 to 1.35)		1.53 (1.38 to 1.70)		2.11 (1.93 to 2.31)		2.35 (2.14 to 2.57)	
Natural direct effect	1.18 (1.06 to 1.32)		1.44 (1.30 to 1.60)		1.91 (1.74 to 2.09)		2.04 (1.86 to 2.24)	
Natural indirect effect	1.02 (1.02 to 1.03)	12	1.06 (1.05 to 1.07)	17	1.11 (1.09 to 1.13)	18	1.15 (1.12 to 1.18)	23
Model 2—adjusted								
Total effect	1.18 (1.05 to 1.31)		1.41 (1.27 to 1.57)		1.72 (1.57 to 1.90)		1.75 (1.58 to 1.94)	
Natural direct effect	1.16 (1.03 to 1.29)		1.36 (1.22 to 1.51)		1.62 (1.47 to 1.79)		1.61 (1.45 to 1.79)	
Natural indirect effect	1.02 (1.01 to 1.02)	11	1.04 (1.03 to 1.05)	14	1.06 (1.05 to 1.08)	14	1.08 (1.06 to 1.11)	18
Long-term sickness absence (9442 events, 8.14%)								
Model 1—crude								
Total effect	1.26 (1.16 to 1.37)		1.52 (1.41 to 1.65)		2.08 (1.94 to 2.22)		2.12 (1.98 to 2.28)	
Natural direct effect	1.24 (1.14 to 1.35)		1.45 (1.34 to 1.57)		1.92 (1.79 to 2.05)		1.90 (1.76 to 2.04)	
Natural indirect effect	1.02 (1.01 to 1.02)	8	1.05 (1.4 to 1.06)	14	1.08 (1.07 to 1.10)	15	1.12 (1.10 to 1.14)	20
Model 2—adjusted								
Total effect	1.21 (1.11 to 1.31)		1.40 (1.29 to 1.52)		1.73 (1.60 to 1.86)		1.66 (1.53 to 1.79)	
Natural direct effect	1.19 (1.09 to 1.30)		1.35 (1.24 to 1.46)		1.64 (1.52 to 1.76)		1.54 (1.42 to 1.67)	
Natural indirect effect	1.01 (1.01 to 1.02)	8	1.04 (1.03 to 1.05)	12	1.06 (1.04 to 1.07)	12	1.07 (1.06 to 1.09)	17
Long-term unemployment (4764 events, 4.11%)								
Model 1—crude								
Total effect	1.33 (1.19 to 1.48)		1.48 (1.32 to 1.64)		1.76 (1.60 to 1.93)		1.57 (1.42 to 1.73)	
Natural direct effect	1.31 (1.18 to 1.47)		1.43 (1.28 to 1.59)		1.66 (1.51 to 1.82)		1.44 (1.30 to 1.60)	
Natural indirect effect	1.01 (1.01 to 1.02)	5	1.03 (1.02 to 1.05)	10	1.06 (1.04 to 1.08)	13	1.09 (1.06 to 1.12)	22
Model 2—adjusted								
Total effect	1.34 (1.20 to 1.49)		1.44 (1.29 to 1.61)		1.60 (1.44 to 1.77)		1.35 (1.21 to 1.51)	
Natural direct effect	1.33 (1.19 to 1.48)		1.42 (1.27 to 1.58)		1.55 (1.40 to 1.72)		1.30 (1.16 to 1.45)	
Natural indirect effect	1.01 (1.00 to 1.01)	3	1.02 (1.01 to 1.03)	6	1.03 (1.01 to 1.05)	8	1.04 (1.02 to 1.07)	15

The reference group consists of individuals with 15 or more years of education (at least 3 years of university).

Crude (model 1) and adjusted (model 2) (RR) with 95% Confidence intervals (CI). Adjusted models are adjusted for all variables measured in childhood and late adolescence. The proportion of the total effect that is due to the indirect effect is presented as proportion mediated (%Δ). The natural direct effect captures the effect of education on the outcome without passing through the mediator, while the natural indirect effect captures the effect of education on the outcome through the mediator.

RRrisk ratio

**Table 4:** Mediation analysis with decomposition of the effect of level of education (in years) and early exit through old-age retirement without income, and early old-age retirement with income, into total effect, direct effect and indirect effect using job control as mediator

Years of education	13–14RR (95% CI)	%∆	12RR (95% CI)	%∆	10–11RR (95% CI)	%∆	≤ 9RR (95% CI)	%∆
Early old-age retirement without income (31 089 events, 28.11%)								
Model 1—crude								
Total effect	1.34 (1.29 to 1.39)		1.44 (1.39 to 1.49)		1.44 (1.40 to 1.49)		1.51 (1.46 to 1.56)	
Natural direct effect	1.34 (1.30 to 1.39)		1.45 (1.40 to 1.50)		1.46 (1.41 to 1.51)		1.53 (1.48 to 1.58)	
Natural indirect effect	0.99 (0.99 to 0.99)	−1	0.99 (0.99 to 0.99)	−2	0.99 (0.98 to 0.99)	−3	0.99 (0.98 to 0.99)	−4
Model 2—adjusted								
Total effect	1.32 (1.28 to 1.37)		1.42 (1.37 to 1.48)		1.42 (1.38 to 1.47)		1.49 (1.44 to 1.55)	
Natural direct effect	1.33 (1.28 to 1.38)		1.43 (1.38 to 1.48)		1.43 (1.38 to 1.48)		1.50 (1.45 to 1.56)	
Natural indirect effect	0.99 (0.99 to 0.99)	−1	0.99 (0.99 to 0.99)	−1	0.99 (0.99 to 0.99)	−2	0.99 (0.99 to 0.99)	−2
Early old-age retirement with income (19 724 events, 17.84%)								
Model 1—crude								
Total effect	1.19 (1.14 to 1.25)		1.14 (1.09 to 1.20)		1.27 (1.22 to 1.32)		1.31 (1.26 to 1.37)	
Natural direct effect	1.18 (1.13 to 1.24)		1.12 (1.07 to 1.18)		1.24 (1.19 to 1.29)		1.26 (1.20 to 1.31)	
Natural indirect effect	1.01 (1.00 to 1.01)	4	1.02 (1.01 to 1.02)	14	1.03 (1.02 to 1.04)	14	1.04 (1.03 to 1.06)	17
Model 2—adjusted								
Total effect	1.18 (1.12 to 1.23)		1.12 (1.07 to 1.18)		1.24 (1.19 to 1.29)		1.27 (1.21 to 1.33)	
Natural direct effect	1.17 (1.11 to 1.22)		1.10 (1.05 to 1.16)		1.20 (1.15 to 1.26)		1.22 (1.16 to 1.28)	
Natural indirect effect	1.01 (1.01 to 1.01)	5	1.02 (1.01 to 1.02)	17	1.03 (1.02 to 1.04)	15	1.04 (1.03 to 1.05)	18

The reference group consists of individuals with 15 or more years of education (at least 3 years of university).

Crude (model 1) and adjusted (model 2) (RR) with 95% Confidence intervals (CI). Adjusted models are adjusted for all variables measured in childhood and late adolescence. The proportion of the total effect that is due to the indirect effect is presented as proportion mediated (%Δ). The natural direct effect captures the effect of education on the outcome without passing through the mediator, while the natural indirect effect captures the effect of education on the outcome through the mediator.

RRrisk ratio

**Table 5:** Mediation analysis with decomposition of the effect of level of education (in years) and early exit through disability pension, long-term sickness absence, long-term unemployment and old-age retirement without income, into total effect, direct effect and indirect effect using physical workload as mediator

Years of education	13–14RR (95% CI)	%∆	12RR (95% CI)	%∆	10–11RR (95% CI)	%∆	≤ 9RR (95% CI)	%∆
Disability pension (5 772 events, 4.98%)								
Model 1—crude								
Total effect	1.21 (1.08 to 1.35)		1.53 (1.38 to 1.70)		2.11 (1.93 to 2.31)		2.35 (2.14 to 2.57)	
Natural direct effect	1.19 (1.07 to 1.33)		1.44 (1.29 to 1.60)		1.90 (1.73 to 2.08)		2.06 (1.87 to 2.26)	
Natural indirect effect	1.01 (1.01 to 1.02)	8	1.06 (1.05 to 1.08)	17	1.11 (1.09 to 1.14)	19	1.14 (1.11 to 1.17)	22
Model 2—adjusted								
Total effect	1.17 (1.05 to 1.31)		1.41 (1.27 to 1.58)		1.73 (1.57 to 1.90)		1.75 (1.58 to 1.94)	
Natural direct effect	1.16 (1.04 to 1.30)		1.34 (1.21 to 1.50)		1.60 (1.45 to 1.76)		1.60 (1.44 to 1.77)	
Natural indirect effect	1.01 (1.01 to 1.02)	8	1.05 (1.04 to 1.07)	17	1.08 (1.06 to 1.10)	18	1.10 (1.07 to 1.12)	20
Long-term sickness absence (9 442 events, 8.14%)								
Model 1—crude								
Total effect	1.26 (1.16 to 1.37)		1.52 (1.41 to 1.65)		2.08 (1.94 to 2.22)		2.12 (1.98 to 2.28)	
Natural direct effect	1.25 (1.15 to 1.36)		1.44 (1.33 to 1.56)		1.88 (1.75 to 2.02)		1.88 (1.74 to 2.02)	
Natural indirect effect	1.01 (1.01 to 1.02)	6	1.06 (1.05 to 1.07)	16	1.11 (1.09 to 1.12)	18	1.13 (1.11 to 1.16)	22
Model 2—adjusted								
Total effect	1.21 (1.11 to 1.31)		1.40 (1.29 to 1.52)		1.73 (1.61 to 1.86)		1.66 (1.53 to 1.79)	
Natural direct effect	1.19 (1.10 to 1.30)		1.33 (1.22 to 1.45)		1.60 (1.48 to 1.73)		1.51 (1.39 to 1.64)	
Natural indirect effect	1.01 (1.01 to 1.02)	7	1.05 (1.04 to 1.06)	17	1.08 (1.06 to 1.10)	18	1.10 (1.08 to 1.12)	22
Long-term unemployment (4 764 events, 4.11%)								
Model 1—crude								
Total effect	1.33 (1.19 to 1.48)		1.48 (1.32 to 1.64)		1.76 (1.60 to 1.93)		1.57 (1.42 to 1.73)	
Natural direct effect	1.32 (1.18 to 1.47)		1.41 (1.26 to 1.57)		1.62 (1.47 to 1.78)		1.41 (1.28 to 1.57)	
Natural indirect effect	1.01 (1.01 to 1.01)	4	1.05 (1.03 to 1.06)	14	1.08 (1.06 to 1.11)	18	1.11 (1.07 to 1.14)	27
Model 2—adjusted								
Total effect	1.34 (1.20 to 1.50)		1.45 (1.30 to 1.62)		1.60 (1.45 to 1.77)		1.35 (1.21 to 1.51)	
Natural direct effect	1.33 (1.19 to 1.48)		1.39 (1.25 to 1.56)		1.51 (36 to 1.67)		1.26 (1.12 to 1.41)	
Natural indirect effect	1.01 (1.01 to 1.01)	4	1.04 (1.02 to 1.05)	12	1.06 (1.04 to 1.08)	15	1.07 (1.05 to 1.10)	26
Early old-age retirement without income (31 089 events, 28.11%)								
Model 1—crude								
Total effect	1.34 (1.29 to 1.39)		1.44 (1.39 to 1.49)		1.44 (1.40 to 1.49)		1.51 (1.46 to 1.56)	
Natural direct effect	1.34 (1.29 to 1.39)		1.43 (1.38 to 1.49)		1.43 (1.39 to 1.48)		1.49 (1.44 to 1.55)	
Natural indirect effect	1.00 (1.00 to 1.00)	0	1.00 (1.00 to 1.01)	2	1.01 (1.00 to 1.02)	3	1.01 (1.00 to 1.02)	3
Model 2—adjusted								
Total effect	1.33 (1.28 to 1.38)		1.43 (1.37 to 1.48)		1.43 (1.38 to 1.48)		1.49 (1.44 to 1.55)	
Natural direct effect	1.32 (1.28 to 1.37)		1.42 (1.37 to 1.47)		1.41 (1.36 to 1.46)		1.48 (1.42 to 1.53)	
Natural indirect effect	1.00 (1.00 to 1.00)	1	1.01 (1.00 to 1.01)	2	1.01 (1.00 to 1.02)	3	1.01 (1.00 to 1.02)	3

The reference group consists of individuals with 15 or more years of education (at least 3 years of university).

Crude (model 1) and adjusted (model 2) (RR) with 95% Confidence intervals (CI). Adjusted models are adjusted for all variables measured in childhood and late adolescence. The proportion of the total effect that is due to the indirect effect is presented as proportion mediated (%Δ). The natural direct effect captures the effect of education on the outcome without passing through the mediator, while the natural indirect effect captures the effect of education on the outcome through the mediator.

RRrisk ratio

### Mediating role of job control

Tables3  4 show the decomposition of the total effect of education on all exit routes into direct effect and indirect effect of job control as a mediator. Low job control mediated the association between level of education and disability pension by 11%–18%, long-term sickness absence by 8%–17%, long-term unemployment by 3%–15% and early old-age retirement with income by 5%–18%, depending on the level of education. However, low job control was not found to mediate the association between education and early old-age retirement without income.

### Mediating role of physical workload

The decomposition of the total effect of educational level on all exit routes into direct effect and indirect effect of physical workload as a mediator is shown in table 5. High physical workload mediated the association between level of education and disability pension by 8%–20%, long-term sickness absence by 7%–22%, long-term unemployment by 4%–26% and early old-age retirement without income by 1%–3%, depending on the level of education.

### Additional analyses

Analysing mediation while excluding those who died or emigrated during the follow-up generated similar results (data not shown) as in the main analysis.

Results from the robustness test, using Cox proportional-hazards regressions, demonstrate similar results compared with the mediation models (online supplemental table 4).

## Discussion

This study demonstrates that educational differences in early labour market exit were partly explained by working conditions. Job control was an important mediator for all exit routes except early old-age retirement without income. Physical workload appears to be an important mediator for all exit routes except early old-age retirement with and without income.

### Job control

In this study, job control explained parts of the association between education and early exit through disability pension, long-term sickness absence and long-term unemployment. This is in line with a majority of previous research that found job control or psychosocial working conditions to partly explain the educational differences in disability pension,^719^,^21^ long-term sickness absence^24^ and long-term unemployment.^18^ Some of the previous research did not find job control to explain educational differences in disability pension^18^ or long-term unemployment^7^ and the different results may be due to differences in measurements or differences in definitions or country-specific regulations. The present study adds to previous literature, which used the same cohort,^20^ by using more contemporary data, including more exit routes and by following the participants until 65 years of age.

We found that job control was an important mediator between the level of education and early old-age retirement with income, but not for early old-age retirement without income. Supporting this finding, previous research found low job control to be associated with a lower risk of leaving the labour market early through old-age retirement without income.^22^ Early old-age retirement without income may be less likely to be related to job control (see table 2) and those exiting through this route may do so more because of other personal or financial reasons.

### Physical workload

In line with previous research,^718^,^21^ we found that high physical workload explained parts of the association between education and early exit through disability pension and long-term unemployment. However, according to a few studies, physical workload did not explain the educational differences in disability pension^18^ and long-term unemployment.^7^ These different results may be explained by the measurement methods and country-specific rules and regulations, as mentioned before. Our finding that physical workload seems to be an important factor for educational differences in long-term sickness absence is strengthened by previous research linking high physical workload to a higher risk of sickness absence, although without examining educational differences in sickness absence.^15 23 28^

This study found physical workload to not be an important factor explaining the educational differences in early exit through early old-age retirement with and without income. These two exit routes could be considered to be more voluntary, unlike disability pension, sickness absence and unemployment, which are often linked to worse health.^7 38^ A potential explanation for these results is that individuals experiencing high physical workload during working life may experience poor health more often than individuals with low physical workload at work. These individuals may, therefore, leave the labour market early through disability pension and sickness absence more often. Previous research found conflicting results regarding the association between high physical workload and the risk of early old-age retirement. High physical workload was not found to be associated with early old-age retirement according to some studies,^7 22^ but on the other hand, one study showed heavy physical workload to be a risk factor for early retirement.^15^ The different results may be explained by different regulations regarding early retirement in different countries or by differences in reasons to retire early, for example, financial or health-related reasons, which may also depend on the educational level of the individual.

### Strengths and limitations

There are several strengths of this study, including the long follow-up, the large cohort and the use of high-quality register data. By including all men who were conscripted for the Swedish military service and using register data, we were able to follow almost all Swedish men up until 65 years of age, expanding previous research. A major strength was the inclusion of five different exit routes, which is particularly valuable given the evolving social security system and differing underlying mechanisms for each exit route. Another strength was the inclusion of early life factors since these seem important for indirect selection into both education and occupation. These factors act as confounders for the relationship between education and early exit and between the working conditions and early exit, and the ability to include these in the models is important to be able to distinguish the working conditions from the early life factors.

Working conditions were measured at the occupational level using two JEMs, allowing us to access job control and physical workload comprehensively. This method reduces the risk of differential misclassification compared with self-reported data, which is a strength. However, it cannot capture all aspects of psychosocial and physical conditions or differences between workers within the same occupation. Furthermore, we estimated the working conditions at one point in time and were unable to account for the conditions prior to this. However, previous studies show that occupational exposures are quite stable over time.^34^ Furthermore, we measured educational level in 2005, which, for most men, was many years after they had completed their education. Therefore, we assume educational level to precede occupational status and working conditions. Moreover, we were not able to disentangle working conditions from later-life health.

Since military conscription was obligatory by law only for men at this time, there are no data for women on factors measured in late adolescence, which is a limitation. The labour market in Sweden is highly gender segregated, and therefore, the exposure to different working conditions and the association between the working conditions and early labour market exit may differ between men and women. However, previous research found educational differences in early labour market exit to be similar for men and women,^8^ even though Swedish women more often receive disability pension and sickness benefits.^39^ Given our definition of long-term sickness absence and long-term unemployment we do not capture a definite early exit, as it is possible for the men to re-enter the labour market. However, a previous study, using less strict definitions of these early exit routes, found that only 30% of the men returned to the labour force.^12^ We were not able to include lifestyle factors measured in adulthood, such as physical activity, food and alcohol intake, which is another limitation since these may influence later-life health.

### Concluding remarks

Low job control and high physical workload at work seemed to explain a substantial part of the educational inequalities seen in early labour market exits of an involuntary kind. However, future studies are needed to fully understand the mechanisms behind the socioeconomic inequalities seen in early labour market exit, especially in relation to early old-age retirement and the role of later-life health. Our results indicate the importance of improving both psychosocial and physical working conditions among older workers to be able to increase later-life labour force participation. This is especially important for the less educated and for not risking increasing inequalities when reforming pension systems and raising retirement ages.

## supplementary material

10.1136/oemed-2024-109594 online supplemental file 1

## Data Availability

Data are available on reasonable request. Data may be obtained from a third party and are not publicly available.
