# Supplementary material for: Mediating effect of working conditions on the association between education and early labour market exit: a cohort study of Swedish men
Source: Occup Environ Med. 2024 Nov 25;81(11):e109594. doi: 10.1136/oemed-2024-109594 (PMC11671893; doi:10.1136/oemed-2024-109594)
Supplement: online supplemental file 1 [file oemed-81-11-s001.pdf]

Supplementary File

The mediating effect of working conditions on the association between education and early labour market exit – a cohort study of Swedish men

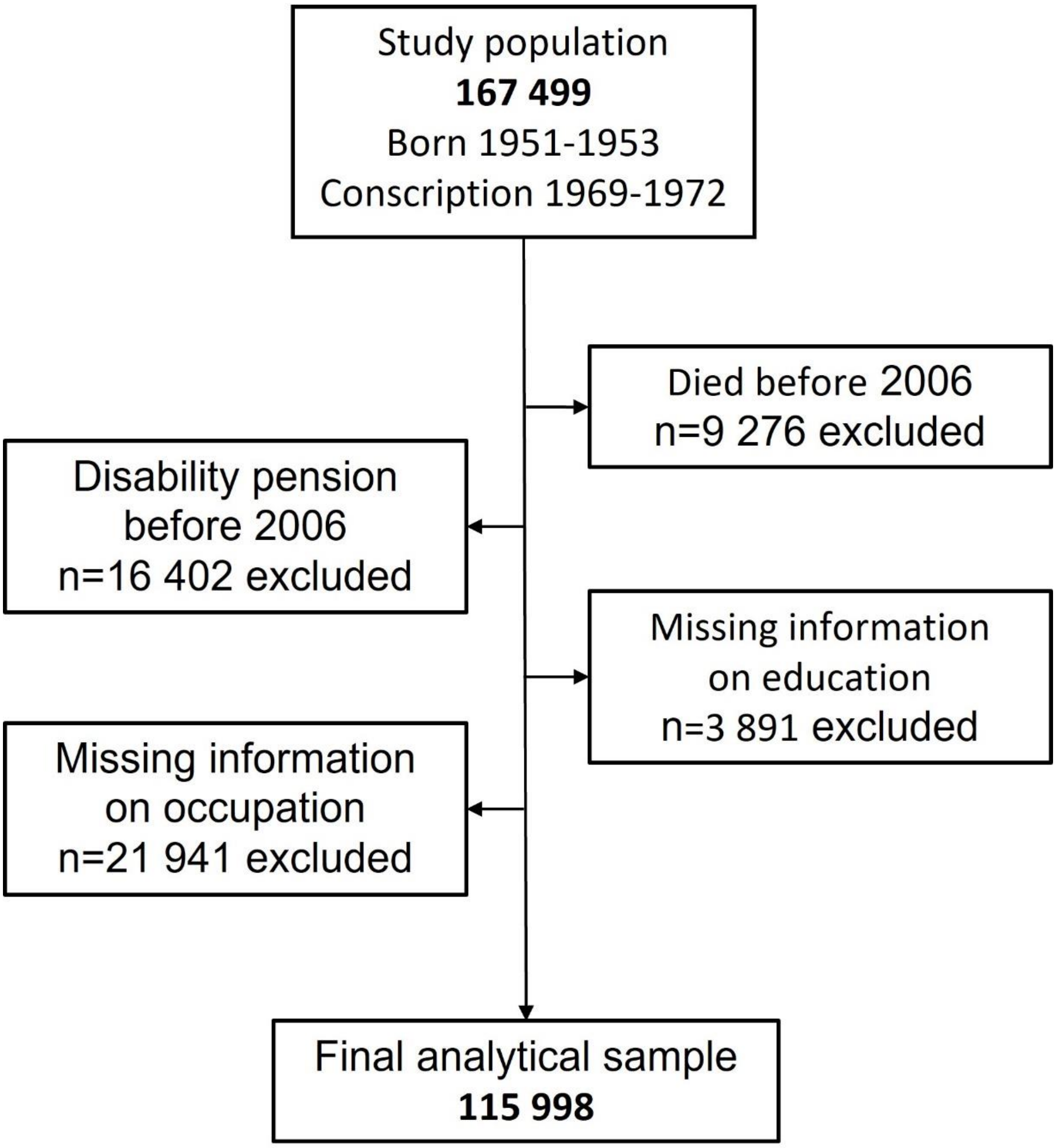

Supplementary Figure 1. Flow chart describing the selection of the participants.

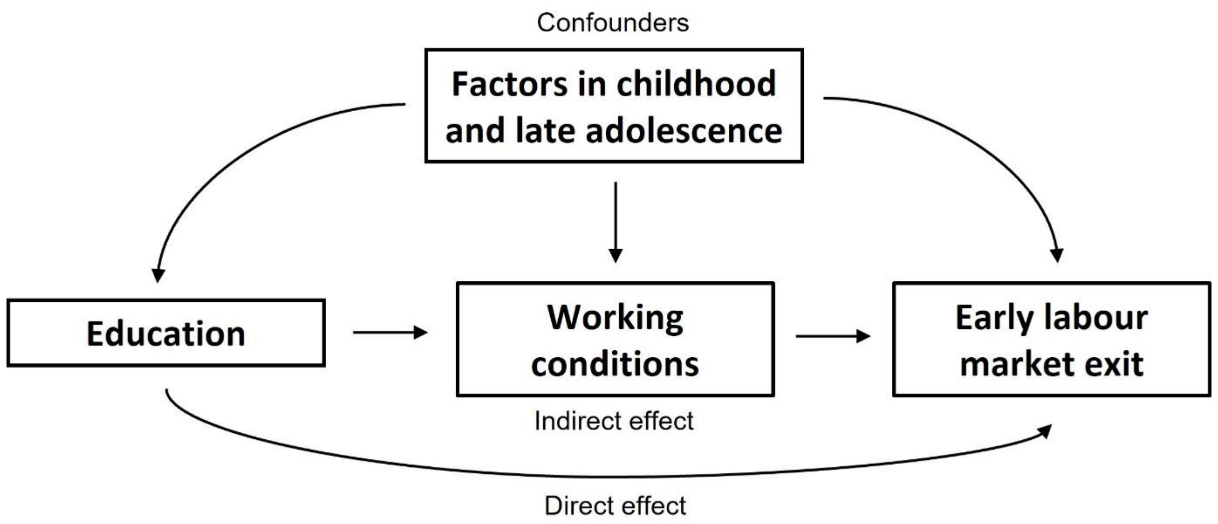

Supplementary Figure 2. Hypothesized model on the relationship between education and early labour market exit, including the direct effect from education to early labour market exit and the indirect effect through the mediating working conditions and the confounding factors measured in childhood and late adolescence.

Description of the variables measured in late adolescence

Cognitive ability was measured on a nine-level scale by an intelligence test including synonyms, induction, spatial capacity, and technical abilities. Stress resilience was rated on a nine-level scale by a psychologist after an interview covering questions on emotional stability, social maturity and coping with stress. For this study, the results on cognitive ability and stress resilience, were categorized as low (1-3), medium (4-6) or high (7-9), using the nine-level scales from the conscription examination. Higher values indicate better functioning. For this study, BMI (weight in kg/height in m2) was dichotomized into normal (<25) or overweight/obese (≥25). All men were examined by a physician, and a psychiatrist if necessary, and psychiatric and musculoskeletal diagnoses were obtained according to the International Classification of Diseases version 8 (ICD-8); 290-315 and 710-738, respectively.

Supplementary Table 1. Baseline characteristics of the individuals included and excluded in the study population.

	Included n (%)	Excluded n (%)	p-value
**Total**	115 988 (69.3)	51 511 (30.7)	
Childhood variable			
Parental education			
≤ 9	68 900 (59.4)	29 057 (56.4)	<0.001
10-11	20 183 (17.4)	8 152 (15.8)	
12	8 842 (7.6)	3 491 (6.8)	
13-14	3 650 (3.2)	1 311 (2.6)	
≥ 15	6 206 (5.4)	2 271 (4.4)	
Missing	8 207 (7.1)	7 229 (14.0)	
Late adolescence variables			
Cognitive ability			
High (7-9)	33 460 (28.9)	9 959 (19.3)	<0.001
Medium (4-6)	57 309 (49.4)	21 846 (42.4)	
Low (1-3)	19 032 (16.4)	11 382 (22.1)	
Missing	6 187 (5.3)	8 324 (16.2)	
Stress resilience			
High (7-9)	27 126 (23.4)	8 339 (16.2)	<0.001
Medium (4-6)	61 214 (52.8)	21 524 (41.8)	
Low (1-3)	21 066 (18.2)	12 973 (25.2)	
Missing	6 582 (5.7)	8 675 (16.8)	
BMI ≥ 25	6 718 (5.8)	3 206 (6.2)	<0.001
Missing	9 808 (8.5)	9 893 (19.2)	
Psychiatric diagnoses	13 797 (11.9)	11 275 (21.9)	<0.001
Musculoskeletal diagnoses	18 589 (16.0)	8 195 (15.9)	>0.05
Years of own education			
≥ 15	21 310 (18.4)	3 612 (7.0)	<0.001
13-14	17 399 (15.0)	3 763 (7.3)	
12	16 898 (14.6)	5 029 (9.8)	
10-11	35 187 (30.3)	13 746 (26.7)	
≤ 9	25 194 (21.7)	12 073 (23.4)	
Missing	0	13 288 (25.8)	

Supplementary Table 2. Complete case analysis excluding 14 877 individuals with missing information on factors measured in childhood and late adolescence. Mediation analysis with decomposition of the effect of level of education (in years) and early exit through disability pension, long-term sickness absence, long-term unemployment, old-age retirement without income, and early old-age retirement with income, into total effect, direct effect and indirect effect using job control as mediator.

**Years of education**	**≥ 15 RR (95% CI)**	**13-14 RR (95% CI)**	**%Δ**	**12 RR (95% CI)**	**%Δ**	**10-11 RR (95% CI)**	**%Δ**	**≤ 9 RR (95% CI)**	**%Δ**
**Disability pension (4 719 events, 4.67%)**									
Model 1 – crude									
Total effect	1.00	1.23 (1.09-1.38)		1.53 (1.36-1.72)		2.14 (1.94-2.36)		2.39 (2.16-2.64)	
Natural direct effect	1.00	1.21 (1.07-1.36)		1.45 (1.29-1.64)		1.96 (1.77-2.16)		2.11 (1.90-2.34)	
Natural indirect effect	1.00	1.02 (1.01-1.02)	10	1.05 (1.04-1.07)	14	1.09 (1.07-1.12)	16	1.13 (1.10-1.16)	20
Model 2 – adjusted									
Total effect	1.00	1.17 (1.03-1.32)		1.38 (1.23-1.56)		1.69 (1.52-1.88)		1.71 (1.53-1.92)	
Natural direct effect	1.00	1.15 (1.02-1.30)		1.33 (1.18-1.50)		1.60 (1.43-1.80)		1.59 (1.41-1.79)	
Natural indirect effect	1.00	1.02 (1.01-1.02)	11	1.04 (1.03-1.05)	14	1.06 (1.04-1.08)	14	1.08 (1.05-1.11)	18
**Long-term sickness absence (7 924 events, 7.84%)**									
Model 1 – crude									
Total effect	1.00	1.32 (1.21-1.45)		1.57 (1.44-1.71)		2.15 (1.99-2.31)		2.25 (2.09-2.43)	
Natural direct effect	1.00	1.30 (1.19-1.42)		1.50 (1.37-1.64)		2.00 (1.84-2.15)		2.03 (1.87-2.20)	
Natural indirect effect	1.00	1.02 (1.01-1.02)	7	1.04 (1.04-1.05)	12	1.08 (1.06-1.09)	14	1.11 (1.09-1.14)	18
Model 2 – adjusted									
Total effect	1.00	1.25 (1.14-1.37)		1.43 (1.30-1.56)		1.77 (1.63-1.92)		1.74 (1.59-1.90)	
Natural direct effect	1.00	1.23 (1.13-1.35)		1.38 (1.26-1.51)		1.68 (1.54-1.82)		1.62 (1.48-1.77)	
Natural indirect effect	1.00	1.01 (1.01-1.02)	7	1.04 (1.03-1.05)	12	1.05 (1.04-1.07)	12	1.07 (1.05-1.09)	16

**Long-term unemployment (3 946 events, 3.90%)**									
Model 1 – crude									
Total effect	1.00	1.33 (1.18-1.50)		1.47 (1.30-1.65)		1.76 (1.59-1.94)		1.58 (1.42-1.76)	
Natural direct effect	1.00	1.31 (1.17-1.48)		1.43 (1.27-1.61)		1.68 (1.52-1.86)		1.49 (1.33-1.67)	
Natural indirect effect	1.00	1.01 (1.00-1.01)	4	1.02 (1.01-1.04)	8	1.04 (1.02-1.07)	10	1.06 (1.03-1.10)	16
Model 2 – adjusted									
Total effect	1.00	1.32 (1.17-1.49)		1.42 (1.26-1.61)		1.58 (1.41-1.77)		1.36 (1.20-1.54)	
Natural direct effect	1.00	1.31 (1.16-1.48)		1.40 (1.24-1.58)		1.55 (1.38-1.73)		1.32 (1.16-1.49)	
Natural indirect effect	1.00	1.01 (1.00-1.01)	3	1.02 (1.00-1.03)	5	1.02 (1.00-1.04)	6	1.03 (1.01-1.06)	12
**Early old-age retirement without income (27 497 events, 28.45%)**									
Model 1 – crude									
Total effect	1.00	1.32 (1.27-1.37)		1.42 (1.37-1.48)		1.43 (1.38-1.48)		1.50 (1.45-1.55)	
Natural direct effect	1.00	1.32 (1.28-1.38)		1.43 (1.38-1.48)		1.44 (1.39-1.49)		1.51 (1.46-1.57)	
Natural indirect effect	1.00	0.99 (0.99-0.99)	-1	0.99 (0.99-0.99)	-1	0.99 (0.99-0.99)	-2	0.99 (0.98-0.99)	-3
Model 2 – adjusted									
Total effect	1.00	1.31 (1.26-1.36)		1.41 (1.36-1.47)		1.41 (1.36-1.46)		1.48 (1.42-1.54)	
Natural direct effect	1.00	1.31 (1.26-1.36)		1.42 (1.36-1.47)		1.42 (1.37-1.47)		1.49 (1.43-1.55)	
Natural indirect effect	1.00	0.99 (0.99-0.99)	-1	0.99 (0.99-0.99)	-1	0.99 (0.99-0.99)	-2	0.99 (0.98-0.99)	-2
**Early old-age retirement with income (17 399 events, 18.00%)**									
Model 1 – crude									
Total effect	1.00	1.18 (1.12-1.24)		1.13 (1.08-1.19)		1.27 (1.22-1.33)		1.32 (1.26-1.38)	
Natural direct effect	1.00	1.17 (1.12-1.23)		1.11 (1.06-1.17)		1.23 (1.18-1.29)		1.26 (1.20-1.32)	
Natural indirect effect	1.00	1.01 (1.01-1.01)	5	1.02 (1.01-1.02)	16	1.03 (1.02-1.04)	15	1.05 (1.03-1.06)	19

Model 2 – adjusted									
Total effect	1.00	1.17 (1.11-1.23)		1.11 (1.06-1.17)		1.24 (1.18-1.30)		1.27 (1.21-1.34)	
Natural direct effect	1.00	1.16 (1.10-1.22)		1.09 (1.04-1.15)		1.20 (1.15-1.26)		1.22 (1.16-1.29)	
Natural indirect effect	1.00	1.01 (1.01-1.01)	5	1.02 (1.01-1.03)	19	1.03 (1.02-1.04)	15	1.04 (1.03-1.05)	18

Crude (model 1) and adjusted (model 2) risk ratios with 95% confidence intervals. Adjusted models are adjusted for all variables measured in childhood and late adolescence. The proportion of the total effect that is due to the indirect effect is presented as proportion mediated (%Δ). The natural direct effect captures the effect of education on the outcome without passing through the mediator, while the natural indirect effect captures the effect of education on the outcome through the mediator.

Supplementary Table 3. Complete case analysis excluding 14 877 individuals with missing information on factors measured in childhood and late adolescence. Mediation analysis with decomposition of the effect of level of education (in years) and early exit through disability pension, long-term sickness absence, long-term unemployment, old-age retirement without income, and early old-age retirement with income, into total effect, direct effect and indirect effect using physical workload as mediator.

Years of education	≥ 15<br>RR (95% CI)	13-14<br>RR (95% CI)	%Δ	12<br>RR (95% CI)	%Δ	10-11<br>RR (95% CI)	%Δ	≤ 9<br>RR (95% CI)	%Δ
**Disability pension (4 719 events, 4.67%)**									
Model 1 – crude									
Total effect	1.00	1.23 (1.09-1.39)		1.53 (1.36-1.72)		2.14 (1.94-2.36)		2.39 (2.16-2.64)	
Natural direct effect	1.00	1.21 (1.07-1.37)		1.45 (1.29-1.63)		1.94 (1.75-2.15)		2.12 (1.91-2.35)	
Natural indirect effect	1.00	1.01 (1.01-1.02)	7	1.06 (1.04-1.07)	16	1.10 (1.08-1.13)	17	1.13 (1.09-1.16)	19
Model 2 – adjusted									
Total effect	1.00	1.17 (1.03-1.33)		1.38 (1.23-1.56)		1.69 (1.52-1.88)		1.72 (1.53-1.93)	
Natural direct effect	1.00	1.16 (1.02-1.31)		1.32 (1.17-1.49)		1.57 (1.41-1.76)		1.58 (1.40-1.78)	
Natural indirect effect	1.00	1.01 (1.01-1.01)	8	1.05 (1.03-1.06)	16	1.08 (1.05-1.10)	17	1.09 (1.06-1.11)	19
**Long-term sickness absence (7 924 events, 7.84%)**									
Model 1 - crude									
Total effect	1.00	1.32 (1.21-1.45)		1.57 (1.44-1.71)		2.15 (1.99-2.31)		2.25 (2.09-2.43)	
Natural direct effect	1.00	1.30 (1.19-1.43)		1.48 (1.35-1.62)		1.94 (1.80-2.09)		1.99 (1.83-2.16)	
Natural indirect effect	1.00	1.01 (1.01-1.02)	5	1.06 (1.05-1.07)	16	1.11 (1.09-1.13)	18	1.13 (1.11-1.16)	21
Model 2 – adjusted									
Total effect	1.00	1.25 (1.14-1.37)		1.43 (1.30-1.57)		1.77 (1.63-1.92)		1.74 (1.59-1.90)	
Natural direct effect	1.00	1.24 (1.13-1.36)		1.36 (1.24-1.49)		1.63 (1.50-1.78)		1.59 (1.45-1.74)	

Natural indirect effect	1.00	1.01 (1.01-1.02)	6	1.05 (1.04-1.07)	17	1.08 (1.07-1.10)	18	1.10 (1.08-1.12)	21
**Long-term unemployment (3 946 events, 3.90%)**									
Model 1 - crude									
Total effect	1.00	1.33 (1.18-1.50)		1.47 (1.30-1.65)		1.76 (1.59-1.94)		1.58 (1.42-1.76)	
Natural direct effect	1.00	1.32 (1.17-1.48)		1.42 (1.26-1.59)		1.65 (1.48-1.83)		1.46 (1.31-1.64)	
Natural indirect effect	1.00	1.01 (1.00-1.01)	3	1.04 (1.02-1.05)	11	1.07 (1.04-1.09)	15	1.08 (1.05-1.12)	21
Model 2 – adjusted									
Total effect	1.00	1.32 (1.17-1.49)		1.43 (1.26-1.61)		1.59 (1.42-1.78)		1.36 (1.20-1.54)	
Natural direct effect	1.00	1.31 (1.16-1.48)		1.38 (1.22-1.57)		1.51 (1.35-1.70)		1.29 (1.13-1.46)	
Natural indirect effect	1.00	1.01 (1.00-1.01)	3	1.03 (1.01-1.05)	10	1.05 (1.02-1.07)	13	1.06 (1.03-1.09)	20
**Early old-age retirement without income (27 497 events, 28.45%)**									
Model 1 - crude									
Total effect	1.00	1.32 (1.27-1.37)		1.42 (1.37-1.48)		1.43 (1.38-1.48)		1.50 (1.45-1.55)	
Natural direct effect	1.00	1.32 (1.27-1.37)		1.42 (1.36-1.47)		1.41 (1.37-1.46)		1.48 (1.43-1.53)	
Natural indirect effect	1.00	1.00 (1.00-1.00)	1	1.01 (1.00-1.01)	2	1.01 (1.00-1.02)	4	1.01 (1.00-1.02)	4
Model 2 – adjusted									
Total effect	1.00	1.31 (1.26-1.36)		1.41 (1.36-1.47)		1.41 (1.36-1.47)		1.48 (1.42-1.54)	
Natural direct effect	1.00	1.31 (1.26-1.36)		1.40 (1.35-1.46)		1.40 (1.35-1.45)		1.46 (1.41-1.52)	
Natural indirect effect	1.00	1.00 (1.00-1.00)	1	1.01 (1.00-1.01)	2	1.01 (1.00-1.02)	3	1.01 (1.00-1.02)	4

Crude (model 1) and adjusted (model 2) risk ratios with 95% confidence intervals. Adjusted models are adjusted for all variables measured in childhood and late adolescence. The proportion of the total effect that is due to the indirect effect is presented as proportion mediated (%Δ). The natural direct effect captures the effect of education on the outcome without passing through the mediator, while the natural indirect effect captures the effect of education on the outcome through the mediator.

Robustness test with Cox proportional-hazards regression

Cox proportional-hazards regressions was used to estimate the association between the level of education and the five different exit routes separately, as well as the association between the confounders and the mediators and early exit, with age as the time scale (see Supplementary Table 4). The assumption of proportional hazards was not violated when tested by Schoenfeld residuals. Since each model was run separately for each outcome, an individual could be observed in several different outcomes. For the outcomes disability pension, long-term sickness absence and long-term unemployment the men were followed from 1 January 2006 until the outcome of interest, emigration, death or until 64 years of age (31 December 2017 at the latest), whichever came first. In Sweden, early old-age retirement can be granted at the earliest at age 61, therefore the men were followed from age 61 (1 January 2012 at the earliest) until the outcome of interest, emigration, death or until 64 years of age (31 December 2017 at the latest), for the outcome early old-age retirement with and without income. Since information on the outcomes came from registers that are updated annually, they do not include exact dates for when the outcome occurred. Therefore, in this study, the day and month for the outcome were set to the middle of the year, the 2nd of July. Adjustments were made in a step-wise order, firstly for all confounding factors only, then job control only, then physical workload only, then job control and all confounding factors simultaneously, and lastly physical workload and all confounding factors simultaneously. The attenuation of hazard ratios (in percent) was calculated using the formula ((HR1-HR2)/(HR1-1))*100. When calculating the attenuation, model 1, 2 and 3 was compared to the crude model and model 4 and 5 was compared to model 1.

Supplementary Table 4. Crude and adjusted hazard ratios (HRs) with 95% confidence intervals (CIs) using Cox proportional-hazards regressions for the association between level of education (in years) and early exit through disability pension, long-term sickness absence, long-term unemployment, old-age retirement without income, and early old-age retirement with income, and percentage of HR reduction (%Δ) by confounders and mediators.

Years of education	≥ 15 HR (95% CI)	13-14 HR (95% CI)	%Δ	12 HR (95% CI)	%Δ	10-11 HR (95% CI)	%Δ	≤ 9 HR (95% CI)	%Δ
**Disability pension (5 772 events, 4.98%)**									
Crude	1.00	1.21 (1.08-1.35)		1.54 (1.38-1.71)		2.12 (1.94-2.32)		2.37 (2.16-2.60)	
Model 1: childhood & late adolescence	1.00	1.17 (1.05-1.32)	16	1.43 (1.28-1.59)	21	1.75 (1.59-1.94)	33	1.80 (1.62-1.99)	42
Model 2: job control	1.00	1.18 (1.06-1.32)	13	1.45 (1.30-1.61)	16	1.91 (1.75-2.10)	19	2.05 (1.86-2.26)	23
Model 3: physical workload	1.00	1.19 (1.06-1.33)	8	1.44 (1.30-1.61)	17	1.90 (1.73-2.09)	20	2.07 (1.88-2.28)	22

Model 4: job control, childhood & late adolescence	1.00	1.16 (1.03-1.30)	8	1.38 (1.23-1.54)	12	1.65 (1.50-1.83)	13	1.66 (1.49-1.84)	17
Model 5: physical workload, childhood & late adolescence	1.00	1.16 (1.04-1.30)	6	1.37 (1.22-1.53)	14	1.63 (1.48-1.80)	17	1.64 (1.48-1.83)	19
**Long-term sickness absence (9 442 events, 8.14%)**									
Crude	1.00	1.26 (1.16-1.38)		1.53 (1.41-1.67)		2.11 (1.97-2.27)		2.16 (2.01-2.32)	
Model 1: childhood & conscription	1.00	1.21 (1.10-1.31)	22	1.40 (1.29-1.53)	25	1.75 (1.62-1.88)	33	1.67 (1.54-1.81)	42
Model 2: job control	1.00	1.24 (1.14-1.35)	8	1.46 (1.35-1.59)	14	1.94 (1.81-2.08)	16	1.92 (1.78-2.07)	21
Model 3: physical workload	1.00	1.25 (1.15-1.36)	6	1.45 (1.33-1.57)	16	1.90 (1.77-2.05)	19	1.90 (1.76-2.05)	23
Model 4: job control, childhood & late adolescence	1.00	1.19 (1.09-1.30)	6	1.36 (1.25-1.48)	11	1.66 (1.53-1.79)	12	1.55 (1.43-1.69)	17
Model 5: physical workload, childhood & late adolescence	1.00	1.20 (1.10-1.30)	5	1.34 (1.23-1.46)	15	1.62 (1.50-1.75)	17	1.53 (1.40-1.66)	21
**Long-term unemployment (4 764 events, 4.11%)**									
Crude	1.00	1.33 (1.19-1.48)		1.48 (1.32-1.65)		1.76 (1.60-1.93)		1.56 (1.41-1.73)	
Model 1: childhood & conscription	1.00	1.33 (1.19-1.49)	-2	1.44 (1.29-1.61)	7	1.60 (1.44-1.77)	21	1.35 (1.20-1.50)	39
Model 2: job control	1.00	1.31 (1.17-1.47)	5	1.43 (1.28-1.59)	10	1.66 (1.51-1.82)	13	1.44 (1.30-1.59)	22
Model 3: physical workload	1.00	1.31 (1.17-1.47)	4	1.41 (1.26-1.57)	14	1.62 (1.47-1.78)	19	1.41 (1.27-1.56)	27
Model 4: job control, childhood & late adolescence	1.00	1.33 (1.18-1.48)	2	1.42 (1.27-1.59)	5	1.55 (1.40-1.72)	7	1.29 (1.15-1.45)	15
Model 5: physical workload, childhood & late adolescence	1.00	1.33 (1.18-1.48)	2	1.40 (1.25-1.56)	10	1.51 (1.36-1.68)	15	1.26 (1.12-1.41)	26

**Early old-age retirement without income (31 089 events, 28.11%)**									
Crude	1.00	1.37 (1.31-1.43)		1.49 (1.43-1.56)		1.48 (1.43-1.54)		1.56 (1.50-1.62)	
Model 1: childhood & conscription	1.00	1.36 (1.30-1.41)	4	1.48 (1.42-1.54)	2	1.47 (1.41-1.53)	2	1.55 (1.49-1.62)	1
Model 2: job control	1.00	1.37 (1.32-1.43)	-1	1.50 (1.44-1.57)	-2	1.50 (1.45-1.56)	-4	1.59 (1.53-1.65)	-5
Model 3: physical workload	1.00	1.37 (1.31-1.43)	0	1.49 (1.43-1.55)	1	1.47 (1.42-1.53)	2	1.55 (1.49-1.61)	3
Model 4: job control, childhood & late adolescence	1.00	1.36 (1.30-1.42)	-1	1.49 (1.42-1.55)	-1	1.48 (1.42-1.54)	-2	1.57 (1.50-1.64)	-3
Model 5: physical workload, childhood & late adolescence	1.00	1.35 (1.30-1.41)	0	1.47 (1.41-1.54)	1	1.46 (1.40-1.52)	3	1.54 (1.47-1.61)	3
**Early old-age retirement with income (19 724 events, 17.84%)**									
Crude	1.00	1.21 (1.15-1.27)		1.16 (1.10-1.22)		1.30 (1.24-1.35)		1.34 (1.28-1.40)	
Model 1: childhood & conscription	1.00	1.19 (1.13-1.26)	8	1.13 (1.07-1.19)	15	1.26 (1.20-1.32)	13	1.29 (1.23-1.36)	13
Model 2: job control	1.00	1.20 (1.14-1.26)	4	1.13 (1.08-1.19)	14	1.25 (1.20-1.31)	14	1.28 (1.22-1.34)	18
Model 3: physical workload	1.00	1.22 (1.16-1.28)	-3	1.18 (1.12-1.25)	-18	1.36 (1.30-1.42)	-20	1.42 (1.35-1.49)	-23
Model 4: job control, childhood & late adolescence	1.00	1.19 (1.13-1.25)	4	1.11 (1.06-1.17)	15	1.22 (1.16-1.28)	14	1.24 (1.18-1.31)	18
Model 5: physical workload, childhood & late adolescence	1.00	1.20 (1.14-1.26)	-2	1.16 (1.10-1.22)	-18	1.31 (1.24-1.37)	-19	1.36 (1.29-1.43)	-21
